# Efficacy of bacteriophages with *Aloe vera* extract in formulated cosmetics to combat multidrug-resistant bacteria in skin diseases

**DOI:** 10.1038/s41598-025-86334-y

**Published:** 2025-02-05

**Authors:** Sodaf A. Maan, Abeer A. Faiesal, Gamar M. Gamar, Noha K. El Dougdoug

**Affiliations:** 1 Department of Agricultural Microbiology, Faculty of Agriculture, Ain Shams University, Cairo, Egypt; 2 Department of Basic and Applied Agriculture Sciences, Higher Institute for Agriculture Cooperation, Cairo, Egypt; 3 Department of Life and Earth Sciences, Higher N’Djamena Institute for Training Teachers, N’Djamena, Chad; 4 Department of Plant and Microbiology, Faculty of Science, Benha University, Banha, Egypt

**Keywords:** *S. aureus*, *P. aeruginosa*, Multidrug-antibiotic resistance bacteria, Cosmetic, *Aloe vera*, Phage therapy, Biological techniques, Biotechnology, Developmental biology, Drug discovery, Microbiology, Health care, Medical research, Pathogenesis

## Abstract

Phage therapy offers a promising alternative to antibiotic treatment for combating illnesses caused by multidrug-resistant bacteria. In this study, pathogenic bacteria *Staphylococcus aureus* and* Pseudomonas aeruginosa* were isolated from pus and skin infected fluidsusing selective media. These bacterial isolates were biochemically identified as *S. aureus* and *P. aeruginosa* with probabilities of 98% and 99%, respectively, through VITEK2 system, and were confirmed as multidrug-resistant based on minimum inhibitory concentration test using colorimetric reagent cards. Lytic phages specific to these isolates were isolated, identified through plaque assays, transmission electron microscopy and classified morphologically according to the new International Committee on Taxonomy of Viruses classification as members of the *Straboviridae, Drexlerviridae,* and *Autographiviridae* families. A cosmetic gel formulation combining *Aloe vera* extract and the phage cocktail was prepared and tested. This gel significantly enhanced phage longevity and reduced bacterial growth by 95.5% compared to the reductions of 90.5% with *Aloe Vera* extract alone and 45.7% with the basic cosmetic gel. The phage remained effective for 4 to over 12 weeks after being preserved in the cosmetic formula, maintaining populations ranging from 5 × 10^3^ to 25 × 10^4^ PFU/mL in vitro. These findings highlight the potential of phage-based formulations, such as Vena Skin Gel, as innovative biotherapeutic tools for managing skin infections.

## Introduction

*Staphylococcus aureus* (*S. aureus*) and *Pseudomonas aeruginosa* (*P. aeruginosa*) are two major pathogens implicated in a variety of skin infections, ranging from mild superficial ailments to severe, life-threatening conditions. The pathogenicity of these bacteria is attributed to their ability to produce a plethora of virulence factors, form resilient biofilms, and develop resistance to multiple antibiotics^1,2^. The emergence of antibiotic-resistant strains has intensified the need for alternative treatment strategies^3,4^.

The use of antibiotics in treating bacterial infections has become increasingly problematic due to the rise of antibiotic resistance^5^. Overuse and misuse of antibiotics have led to the emergence of multidrug-resistant bacteria.This resistance has made bacterial infections increasingly difficult to treat, contributing to prolonged—hospital stays, higher medical costs, and increased mortality. Furthermore, antibiotics can-disrupt the natural microbiota, leading to secondary infections and other health issues^6,7^. As the effectiveness of traditional antibiotics diminishes, alternative treatments are urgently needed to address these challenges.

Bacteriophages, or phages, are viruses that specifically infect and lyse bacterial cells. Phage therapy, which involve using bacteriophages to treat bacterial infections, has garnered renewed interest as an alternative to traditional antibiotics, particularly in response to rising antibiotic resistance^8,9^. Phages are highly specific to their bacterial hosts, allowing them to target pathogenic bacteria without disturbing beneficial microbiota. Phages can also evolve alongside bacteria, potentially overcoming bacterial resistance. Moreover, phage therapy can be tailored to individual bacterial strains, offering a personalized approach to treatment^10–12^.

Phage therapy has shown promise in treating infections caused by antibiotic-resistant bacteria, including Methicillin-resistant *Staphylococcus aureus* (MRSA) and *P. aeruginosa*^13,14^. Phage function through a lytic cycle, during which they infect bacterial cells, replicate within the host, and eventually cause lysis of the host cell to release progeny phages, thereby reducing bacterial populations^15,16^. Phages can also disrupt biofilms by degrading the extracellular matrix and killing the bacteria within, making them an effective tool against biofilm-associated infections^17,18^.

*Aloe vera*, a succulent plant, has been used for centuries for its medicinal properties, including its antimicrobial activity. *Aloe vera* gel contains a variety of bioactive compounds such as anthraquinones, saponins, and polysaccharides, which have been shown to possess antibacterial properties^19,20^. These compounds can disrupt bacterial cell walls, inhibit enzyme activity, and interfere with the replication of bacterial DNA. Studies have demonstrated that *Aloe vera* extracts exhibit antibacterial activity against a range of pathogens, including *S. aureus* and *P. aeruginosa*. The gel’s antimicrobial effects are attributed to compounds such as aloin, emodin, and acemannan, which can penetrate bacterial cells and disrupt their functions^21,22^. *Aloe vera* also has anti-inflammatory and wound-healing properties, making it a valuable adjunct in the treatment of skin infections^23–25^.

The combination of phage therapy and *Aloe vera* extract offers a synergistic approach to combating multidrug-resistant bacterial infections. Phages specifically target and lyse pathogenic bacteria, while *Aloe vera* enhances this effect through its broad-spectrum antimicrobial properties and ability to promote wound healing. This combined approach can potentially overcome the limitations of antibiotic therapy and provide a more effective treatment for stubborn skin infections caused by *S. aureus and P. aeruginosa*.

Bacteriophage therapy and *Aloe vera* extract represent promising alternative treatments that can enhance antibacterial activity and improve clinical outcomes. Their combined use could lead to more effective management of skin infections, particularly those caused by multidrug-resistant pathogens.

## Material and methods

### Collection and extraction of *Aloe vera* leaves

*Aloe vera* leaves were obtained from the Horticulture Department (branch of medicinal and aromatic plants) at the Faculty of Agriculture, Ain Shams University, Cairo, Egypt, where they are permanently cultivated. This process was conducted with the permission of the Experimental Plant Care and Research Ethics Committee at Ain Shams University and the Agriculture Sector Committee. The leaves were surface cleaned by gently scrubbing with a wet toothbrush and rinsing under cold running water. The cleaned leaves were then cut to remove the base, spines, and skin using a sharp kitchen knife. The gel was stored in a plastic container away from direct sunlight^26^. After air drying, 50 g of leaves were macerated in a warning blender for 10 min with 100 mL of sterile distilled water. The macerate was then filtered through two layers of muslin cloth and centrifuged at 4000×*g* for 30 min. The supernatant liquid was heat sterilized and further filtered using Wattman No.1 filter paper. Finally, the extract was stored aseptically at 5 °C.

### Preparation of cosmetic gel

*Aloe vera* gel was prepared by mixing the following ingredients: 8% stearic acid, 4% stearyl alcohol, 3% cetostearyl alcohol, 5% glycerin, 5% paraffin oil, 3% isopropyl myristate, 5% Tween 20, 0.5% active ingredient, 7% perfume, and 1% methyl paraben. Sodium hydroxide was added dropwise to this mixture along with 0.1–0.5% *Aloe vera*. Separately, carbopol was completely dissolved in 100 mL of distilled water, and then sodium hydroxide was added dropwise with stirring to form the gel. Finally, *Aloe vera* was added using a vortex mixer^27^.

### Collection of clinical samples

Clinical swabs were collected from patients with edema, erythematic furuncles, and vesicles that produced pus and fluid at the dermatology hospital in Qalyubia governorate. These swabs were placed in nutrient broth tubes and incubated at 37 °C until used. This procedure was conducted in accordance with relevant institutional, national, and international guidelines and legislation, as approved by the Research Ethics Committee at Ain Shams University, Agriculture Sector Committee.

### Isolation and identification of pathogenic bacteria

The nutrient broth containing clinical swabs was diluted in broth medium and inoculated onto cetrimide agar (Oxoid; CM559) for *P. aeruginosa* and Baird Parker media (Oxoid; CM0275) for *S. aureus*. The inoculated plates were incubated at 37 °C for 48 h. The isolated cultures were identified using Gram staining, and the minimal inhibitory concentration (MIC) of selected antibiotics was determined using colorimetric reagent cards from BioMérieux, France. These cards were incubated and automatically read by the VITEK2 system.

### Isolation and identification of bacteriophages

Bacteriophages were isolated from sewage water from the Dermatology hospital in Benha City, Egypt, using the enrichment technique^28^. Sewage water samples were centrifuged at 13,000 rpm for 20 min and then filtered through a sterile Millipore filter (0.45 µm). The filtrate (50 µL) was added to an equal volume of nutrient broth inoculated with a mixture of *S. aureus* and* P. aeruginosa* isolates at log phase (4 × 10^6^ and 2 × 10^7^ CFU/mL, respectively)^29^. The flask was incubated with shaking at 37 °C for 48 h. The broth cultures were centrifuged at 6000 rpm for 15 min, and the supernatant was sterilized by passing it through a 0.45 µm Millipore membrane to be used as a phage reservoir. Using the double agar overlay technique^28^, *S. aureus* and* P. aeruginosa* bacterial lawns were cultured on nutrient agar plates by adding 200 µL of tested bacteria (OD of 0.4 at 600 nm) to 4 mL semi-solid nutrient agar and pouring the mixture over nutrient agar plates. 10 µL of bacteriophage sources were then deposited on bacterial lawns and allowed to dry. Plates were incubated overnight at 37 °C to develop lytic spots.

### Purification and propagation of phages

Single plaque assay was performed using a sterile pasture pipette to isolate bacteriophages^28^. A single plaque was removed and incubated at 37 °C with shaking at 1200 rpm in 0.5 ml of nutrient broth containing 100 μL of *S. aureus* for one test and *P. aeruginosa* for another. The mixture was centrifuged at 10,000×*g* for 10 min. The bacteria were removed by filtering the supernatant through a 0.45 μm Millipore membrane. The phage combinations were stored at 4 °C in 2 mL Eppendorf tubes.

### Biological characterization of phages

#### pH stability

The isolated bacteriophages (10^8^ PFU/mL) were suspended in CM phage buffer (0.735 g/l CaCl_2_·2 H_2_O: 2.5 g/l MgSO_4_·7 H_2_O; 0.05 g/l Gelatin; 6 ml/l 1 M Tris buffer; pH 7.2) at pH levels 4, 5, 6, 7, 8, 9, and 10 and incubated at 37 °C for 30 min. Phage effectiveness was assessed using double-layer plaque assay technique with *S. aureus* and *P. aeruginosa* isolates as indicator hosts^30^.

#### UV stability

To test UV sensitivity, 10 mL of isolated bacteriophages (10^8^ PFU/mL) were placed in a petri-dish at distances of 15, 30, and 45 cm from a 30-Watt UV electric lamp, 240 nm (Ushio, Japan). All experiments were conducted at night to avoid additional UV exposure. The exposure times were 30, 60, 90, and 120 min. Phage activity was assessed using double-layer plaque assay technique^30^ with *S. aureus* and *P. aeruginosa* isolates as indicators.

#### Bacteriophage morphology

Purified phage samples (10^7^ PFU/mL) were negatively stained with 1% aqueous uranyl acetate and examined using a transmission electron microscope (JEOL-JEM-1010 TEM) at the Regional Center for Mycology, Al-Azhar University.

#### Infectivity of phages

*Staphylococcus aureus* and* P. aeruginosa* were diluted in sterile distilled water to a density of 10^6^ CFU/mL and inoculated onto nutrient agar plates. Drops (About 30 µL) of each isolated phage (approximately 10^8^ PFU/mL) were applied to the cultured agar plates. Plates were incubated overnight at 37 °C. Clear or turbid confluent lysis was considered a positive result, while extremely faint zones were considered negative^31^.

#### Cross infectivity of phages

To determine the cross infectivity of phages, bacterial isolates listed in Table 4 were used. Each bacterial host at stationary phase (200 µL) was mixed with 0.6% soft agar and spread on LB plates. Tenfold serially diluted phage lysate (2.5 µL) was spotted on the bacterial lawn. Plates were incubated overnight at 37 °C. The presence and size of plaques or clear zones were documented, indicating the lytic activity of the phages^32^.

### Determination of antibacterial activity of phages and formulated cosmetic

#### Preparation of phages and cosmetic formulation

**Fig. 1:**
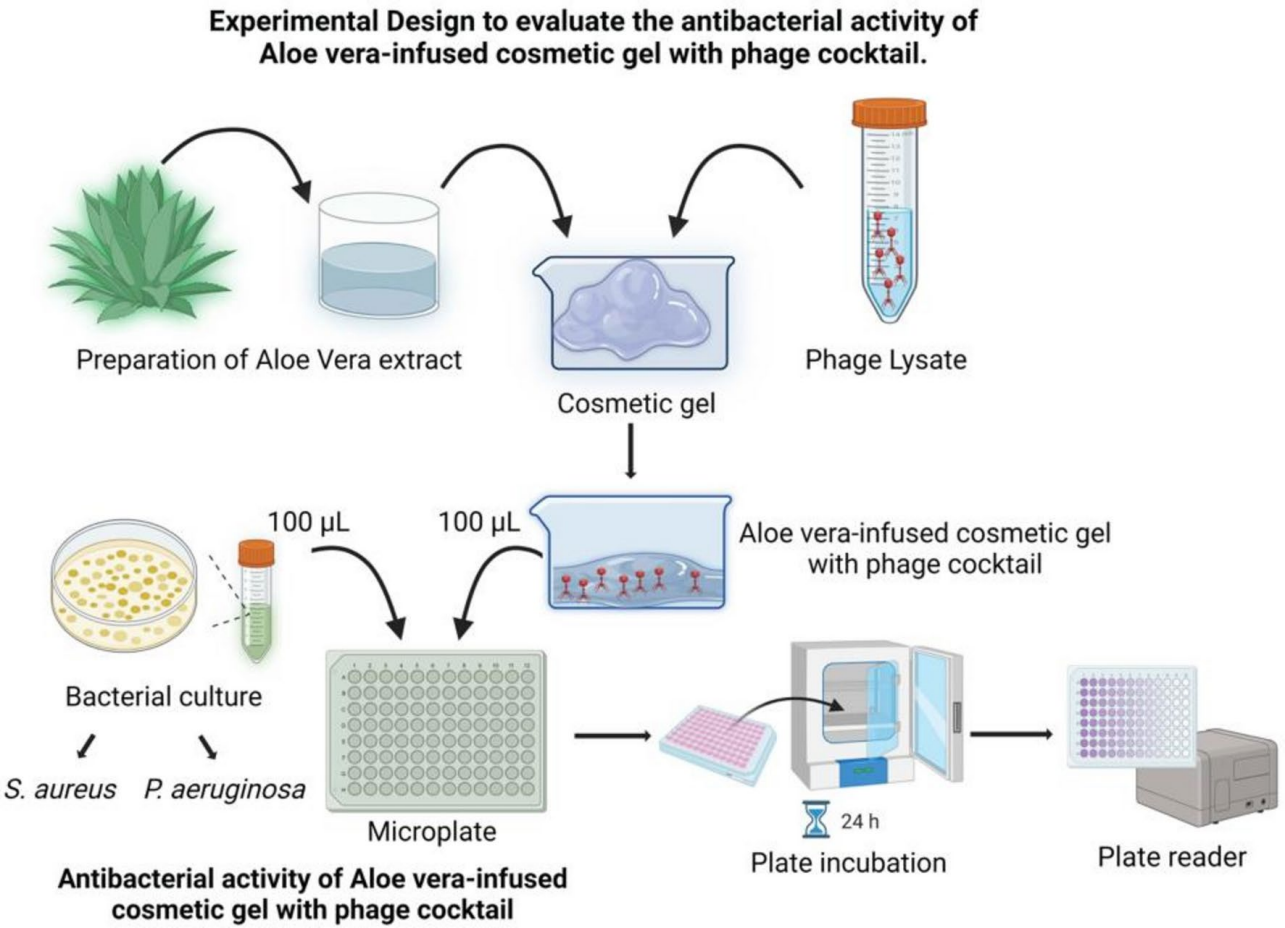
Flow diagram outlining the experimental process for assessing the antibacterial activity of a cosmetic gel infused with *Aloe vera* extract and a phage cocktail. The *Aloe vera* extract was prepared and combined with phage lysate, then 100 µL of this mixture was applied to microtiter plates on a bacterial lawn. Comparisons were made between the formulated gel, phages alone, and *Aloe vera* extract alone. After incubation, bacterial growth was measured at 620 nm OD using an ELISA microplate reader. Results, expressed as means ± SE from three replicates. This image was created with BioRender.com.

The formulated Cosmetic gel was prepared as shown in Fig. 1 by emulsifying 5 g of CG with 5 mL of *Aloe vera* extract (AVE) and 5 mL of phage cocktail (PC) at a concentration of 10^8^ PFU/mL. The resulting formulation, named as F(CGAVEPC), consists of Cosmetic gel (CG) + *Aloe vera* extract (AVE) + Phage cocktail (PC). The formulated CG was stored in an airtight plastic container away from direct sunlight^20^.

#### Longevity of formulated cosmetic gel, F(CGAVEPC)

The phage cocktail (PC) alone and mixed with each component of the formulated CG (CG and AVE) were preserved in 2 mL Eppendorf tubes. Samples were stored at room temperature and at 4°C (refrigerator) for 7, 15, 30, 60, and 90 days. The survival of phages was assessed using double-layer plaque assay technique, utilizing *S. aureus* and* P. aeruginosa* as indicator hosts^30^.

#### Antibacterial activity test

The antibacterial activity against *S. aureus* and* P. aeruginosa* was evaluated using three treatment categories: phages only, CG only, and formulated CG with phages. Bacterial reduction was determined using the microplate dilution method (NCCLS/CLSI, 2007). Bacterial suspensions were prepared to a turbidity equivalent to 0.5 McFarland standards (10^8^ CFU/mL) in Mueller Hinton Broth (MHB) and then diluted to 10^6^ CFU/mL. 100 µL of each bacterial suspension was added to 15 wells of a polystyrene tissue culture plate. 100 µL of phage populations, CG, and formulated cosmetic with phages were added to the wells (Fig. 1). Three well replicates were used for each treatment: untreated bacteria (control), standard antibiotics (Ampicillin for *S. aureus* and Gentamicin for *P. aeruginosa*), and media only (background control). After 24 h of incubation at 37°C, bacterial growth was measured at 620 nm OD using an ELISA microplate reader. The percentage of bacterial growth reduction (% GR) was calculated using the formula: %*GR* = (*C* − *TC*) × 100%*GR* = (*CC* − *T*) × 100 where C is the number of untreated cells, and T is the number of treated cells. Results were recorded as means ± SE of three replicates.

#### Antibiofilm test

Biofilm formation and inhibition were assessed using the quantitative Tissue Culture Plate Technique^33^. TSB medium inoculated with *S. aureus* and *P. aeruginosa* was dispensed into ELISA plates (200 µL/well in 3 replicates). 50 µL of phage populations (5 × 10^6^ PFU/mL), CG, and formulated cosmetic with phages were added to the wells. Positive control wells contained tested bacteria without treatment, and negative control wells contained only TSB broth medium. Plates were incubated at 37 °C for 24 h. After incubation, plates were washed three times with 2.0 mL phosphate buffer (pH 7.2), fixed with 2% sodium acetate, and stained with 0.1% crystal violet. Plates were cleaned with distilled water, dried, and the optical density of cell viability and biofilm samples was measured at 620 nm and 570 nm, respectively, using an ELISA reader. The study was conducted three times with consistent results.

#### Statistical analysis

The experiments were conducted in three separate tests. Antibacterial activity data were statistically evaluated using SPSS for Windows 16.0. Differences among means were tested at a significance level of *P* < 0.05. Results were considered statistically significant if *P* < 0.05, with distinct superscripts (a, b) indicating significant differences (*P* < 0.05).

## Results

### Identification of pathogenic isolated bacteria

On cetrimide agar medium, *Pseudomonas aeruginosa* colonies exhibited a blue-green pigment called pyocyanin, with light outer edges and greenish-black centers, measuring 0.8–2.2 mm in diameter.* Staphylococcus aureus* colonies on Baird Parker agar appeared gray-black, glossy, and convex, with a white boundary and a clearing zone around them. *P. aeruginosa* cells were straight rods and Gram-negative, whereas *S. aureus* cells were cocci in clusters and Gram-positive. The biochemical characteristics of *P. aeruginosa* and *S. aureus* were confirmed with excellent probabilities of 98% and 99%, respectively, after full biochemical identification using the VITEK2 system. Both bacterial isolates were found to be multidrug-resistant based on minimum inhibitory concentration (MIC) testing with VITEK2 colorimetric reagent cards, which were interpreted automatically.

### Isolation and characterization of phage

**Fig. 2:**
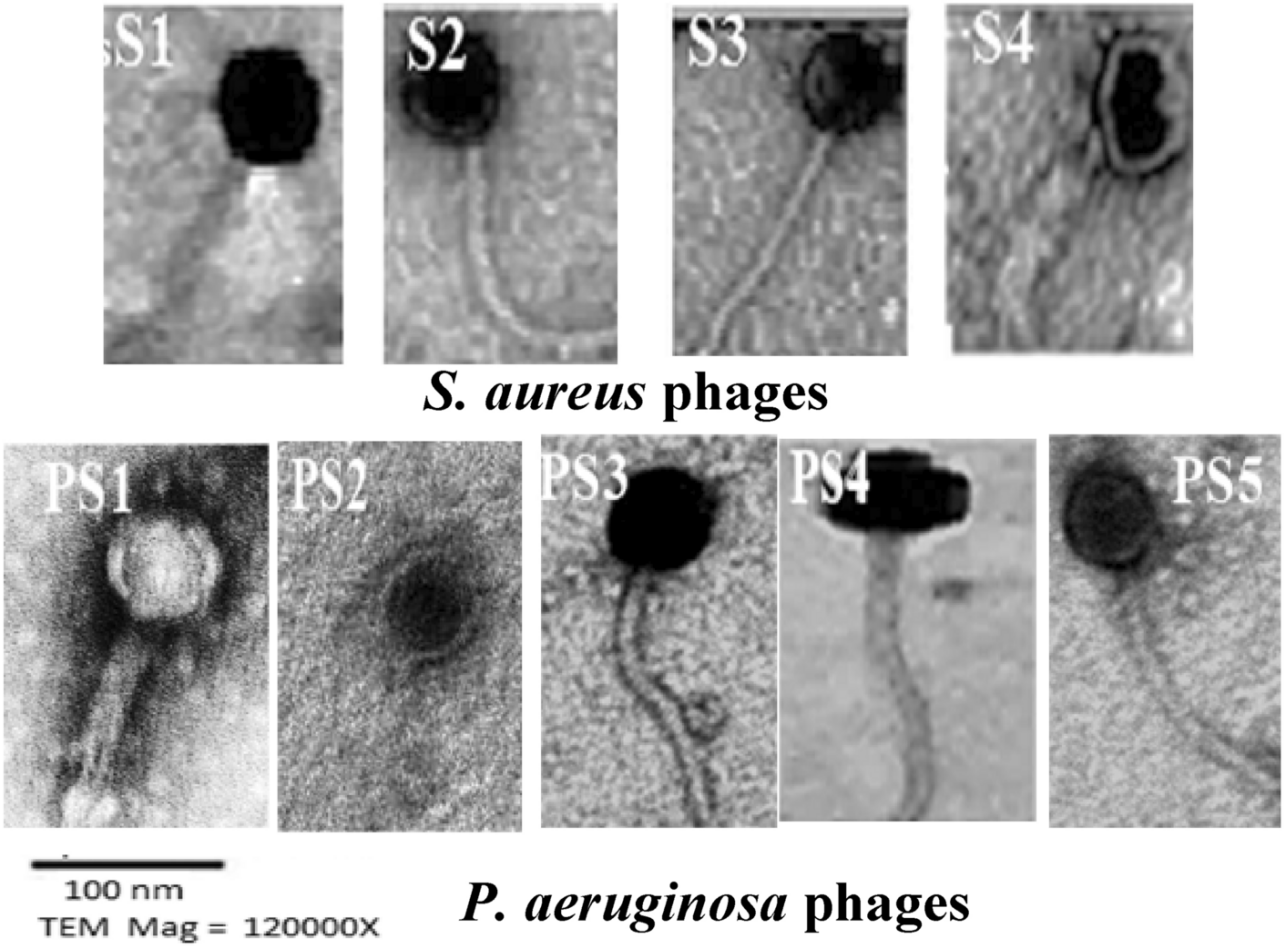
Electro-micrograph represents the morphology of nine different bacteria viruses specific to *S. aureus* and *P. aeruginosa* isolates using TEM.

Nine isolated phages were isolated and exhibited varying plaque morphologies, ranging from clear to turbid confluent lysis and small to large circular sizes of 3–5 nm. These phages were classified Morphologically into the families *Straboviridae, Drexlerviridae,* and *Autographiviridae* based on the new ICTV classification (Fig. 2 and Table 1). The titer of the nine phage isolates was approximately 1 × 10^8^ PFU/mL. The phage particles displayed different morphotypes, including short to long, curled, non-contractile tails measuring 200.3–245.5 nm in length and 15.4–18.5 nm in width, with heads varying in size from 65.2 to 75.5 nm (Fig. 2).

**Table 1:** Dimensions and taxonomy of phages specific to *S. aureus* and *P. aeruginosa* isolates based on new ICTV classification.

Phages	Head diameter (nm)	Tail length (nm)	Family
S1	84.0	182.28	*Straboviridae*
S2	96.00	164.78	*Autographiviridae*
S3	123.00	121.41	*Autographiviridae*
S4	132.00	165.33	*Straboviridae*
Ps1	93.00	145.60	*Straboviridae*
Ps2	95.00	186.40	*Drexlerviridae*
Ps3	65.00	197.20	*Autographiviridae*
Ps4	75.00	178.10	*Autographiviridae*
Ps5	85.00	208.10	*Autographiviridae*

### Biological characters of isolated phages

The lytic activity of the nine phage isolates that targeting both *S. aureus* and *P. aeruginosa* were assessed by a trauma specialist and eight bacteria reference labs. *S. aureus* isolates exhibited variable sensitivity to individual *S. aureus* phages, while *P. aeruginosa* isolates showed variable sensitivity to individual *P. aeruginosa* phages, each at a final titer of 10^6^ PFU/mL. The phage cocktail (PC) at a final titer of 10^8^ PFU/mL infected all eight bacteria reference labs. Four *S. aureus* phages did not affect four *P. aeruginosa* isolates, and similarly, five *P. aeruginosa* phages did not affect four *S. aureus* isolates. Positive results were indicated by clear or turbid confluent lyses, while extremely faint zones indicated negative results (Table 2).

**Table 2:** The infectivity of isolated bacteriophages to different *S. aureus *and* P. aeruginosa* isolates by double layer technique.

Tested host	Phage isolates									
	*S. aureus*				*P. aeruginosa*					PC
	S1	S2	S3	S4	Ps1	Ps2	Ps3	Ps4	Ps5	
*S. aureus* Ref-iso	7.12a	–	7.40a	0.95d	–	–	–	–	–	7.99a
*S. aureus* Ref-Lab 1	–	7.34a	–	7.00a	–	–	–	–	–	7.19a
*S. aureus* Ref-Lab 2	6.91b	–	6.87a	–	–	–	–	–	–	6.98b
*S. aureus* Ref-Lab 3	–	6.23b	7.34a	7.87a	–	–	–	–	–	7.33a
*P. aeruginoia* Ref-iso	–	–	–	–	7.12a	7.34a	0.84d	6.97	7.11a	7.45a
*P. aeruginoia* Ref-Lab	–	–	–	–	–	–	5.99c	–	–	6.19b
*P. aeruginoia* Ref-Lab	–	–	–	–	–	6.87b	6.38b	0.69d	–	6.95b
*P. aeruginoia* Ref-Lab	–	–	–	–	6.23b	0.35d	–	7.15a	6.98b	7.13a

S1-S2, *S. aureus* phages; Ps1-Ps5, *P. aeruginosa* phages; PC, phage cocktail.

a–d: Differences between the study sites indicated by different letters are statistically significant (*P* < 0.05). Treatments with different letters are significantly different from each other, with “a” representing the Control group.

The nine phage isolates targeting *S. aureus* and *P. aeruginosa* exhibited stability across a range of pH values (4, 5, 6, 7, 8, 9, and 10) at 37 °C for 30 min. Phage survival was assessed through double-layer plaque assay technique in vitro, utilizing *S. aureus* and* P. aeruginosa* isolates as indicator hosts (Table 3).

**Table 3:** Phages sensitivity to pHs using *S. aureus* and *P. aeruginosa* as an indicator host.

pH test		Phage isolates of *S. aureus*			Phage isolates of *P. aeruginosa*						
		Initial titer of phage is 7.91a (log PFU/ml)									
		S1	S2	S3	S4	Ps1	Ps2	Ps3	Ps4	Ps5	PC
pH	4	–	–	–	–	–	–	–	–	–	1.09d
	5	–	3.50c	–	–	–	–	–	–	–	3.91c
	6	6.11b	6.20b	5.73b	6.09b	6.90b	6.08b	6.30b	5.81b	5.92b	6.15a
	7	7.90a	7.87a	7.95a	7.77a	7.90a	7.84a	7.90a	7.99a	7.84a	7.99a
	8	6.11b	6.20b	5.73b	6.09b	6.90b	6.08b	6.30b	5.81b	5.92b	7.15a
	9	–	–	3.90c	–	–	3.92c	3.87c	–	–	3.12c
	10	–	–	–	–	–	–	–	–	–	1.21d

S1-S2, *S. aureus* phages; Ps1–Ps5, *P. aeruginosa* phages; PC, phage cocktail.

a–d: Differences between the study sites indicated by different letters are statistically significant (*P* < 0.05). Treatments with different letters are significantly different from each other, with “a” representing the Control group.

The nine phage isolates targeting *S. aureus* and *P. aeruginosa* were found to be stable when exposed directly to UV irradiation at approximately 45 cm for 120 min. All manipulations were conducted in the dark to prevent UV irradiation interference, and the stability was assessed using double-layer plaque assay technique against *S. aureus* and* P. aeruginosa* isolates as indicator hosts (Table 4).

**Table 4:** Phages sensitivity to U.V. by double layer technique.

UV exposure		Phage isolates of *S. aureus*				Phage isolates of *P. aeruginosa*					
		S1	S2	S3	S4	Ps1	Ps2	Ps3	Ps4	Ps5	PC
		Initial titer: 7.38a (log PFU/ml)									
15 cm	30 min	5.17b	5.07b	5.13b	5.22b	5.18b	5088b	5.23b	5.11b	5.25b	5.79b
	60 min	2.57c	2.58c	3.12c	2.97c	2.57c	3.07c	2.98c	2.27c	2.99c	3.99c
	90 min	–	–	–	–	–	–	–	–	–	–
	120min	–	–	–	–	–	–	–	–	–	–
30 cm	30 min	5.9b	5.77b	5.73b	5.81b	5.9b	5.99b	5.6b	5.84b	5.53b	6.95a
	60 min	3.57c	2.99c	3.12c	2.97c	3.57c	3.07	2.97	3.27	2.99	5.98b
	90 min	1.67d	1.69d	1.6d	1.59d	1.81d	1.93d	1.74d	1.65d	1.5d	3.19c
	120min	–	–	–	–	–	–	–	–	–	–
45 cm	30 min	7.19a	7.21a	6.99a	7.14a	7.02a	7.25a	7.18a	7.05a	7.18a	7.49a
	60 min	6.91a	6.77a	6.63a	6.81a	6.90a	6.99a	7.06a	6.84a	6.95a	7.03a
	90 min	5.77b	5.71b	5.63b	5.51b	5.77b	5.88b	5.53b	5.51b	5.84b	5.91b
	120min	3.57c	3.57c	3.12c	3.97c	3.57c	3.07c	3.97c	3.27c	3.99c	5.38b

S1-S2, *S. aureus* phages; Ps1–Ps5, *P. aeruginosa* phages; PC, phage cocktail.

a–d: Differences between the study sites indicated by different letters are statistically significant (*P* < 0.05). Treatments with different letters are significantly different from each other, with “a” representing the Control group.

### Antibiofilm activity of formulated cosmetic gel F (CG + AVE + PC)

The effects of cosmetic gel (CG), *Aloe vera* extract (AVE), phage cocktail (PC), and their combined formulation on cell viability and biofilm formation were assessed (Table 5). Significant reductions in cell viability were observed, with reductions of 18.27%, 76.69%, 91.74%, and 97.11% for *S. aureus* and 6.02%, 62.31%, 92.07%, and 93.77% for *P. aeruginosa* isolates when treated with CG, AVE, PC, and the formulated F(CG + AVE + PC), respectively. Additionally, the formulated cosmetic gel (FCGAVEPC) exhibited strong antibiofilm activity compared to its individual components. The ingredients of the formulated CG significantly reduced biofilm formation by 18.93%, 79.65%, 86.26%, and 88.90% for *S. aureus* and by 24.48%, 72.90%, 81.56%, and 87.46% for *P. aeruginosa* isolates when treated with CG, AVE, PC, and the formulated F(CG + AVE + PC), respectively.

**Table 5:** Biofilm formation assay and biofilm reduction (%) of *S. aureus* and* P. aeruginosa* isolates using (ELISA plate method).

Assessments		Treatments									
		*S. aureus*					*P. aeruginosa*				
		CG	AVE	PC	FCAP		CG	AVE	PC		FCAP
Cell viability (OD 620 nm)	Pre	1.866a					1.526a				
	Post	1.525a	0.435b	0.154c		0.054d	1.434a	0.575b		0.121c	0.095d
% Reduction of Cell viability		18.27a	76.69b	91.74c		97.11d	6.02a	62.31b		92.07c	93.77d
NC (OD.620nm)		0.05a					0.05a				
Biofilm (OD 570 nm)	Pre	0 .757a					0.678a				
	Post	0.615a	0.154b	0.104c		0.084d	0.512a	0.184b		0.125c	0.085d
% Biofilm reduction		18.93a	79.65b	86.26c		88.90d	24.48a	72.90b		81.56c	87.46d

CG, cosmetic gel; PC, phage cocktail; AVE, *Aloe vera* extract; FCAP, formulated cosmetic gel + *Aloe vera* extract + Phage cocktail; NC, negative control (nutrient broth).

a–d: Differences between the study sites indicated by different letters are statistically significant (*P* < 0.05). Treatments with different letters are significantly different from each other, with “a” representing the Control group.

### Longevity of formulated bacteriophages

The formulated phage cocktail showed efficacy against both *S. aureus* and *P. aeruginosa* isolates. Its effectiveness persisted when mixed with CG for one month under room and cooling temperature. Moreover, it maintained its effectiveness for two months when mixed with AVE and for three months when mixed with CG + AVE at room and 4 °C temperature (Table 6).

**Table 6:** Phages viabilities stored longevity at different time by double layer technique.

Type and time preservation		Forms of phage preservation			
		PC	CG + PC	AVE + PC	F(CGAVEPC)
Room temperature	7 days	7.49a	7.83a	7.79a	7.55a
	15 days	5.61b	5.72b	7.92a	7.94a
	30 days	3.46c	3.96c	5.69b	5.76b
	60 days	2.15d	–	3.47c	4.08c
	90 days	–	–	–	2.41d
4 °C (Refrigerator)	7 days	7.79a	7.82a	7.83a	7.5a
	15 days	5.64b	5.58b	5.73b	7.49a
	30 days	3.63c	5.64b	5.69b	5.53b
	60 days	3.54c	3.46c	5.27b	5.37b
	90 days	–	–	2.91d	4.19c

CG, cosmetic gel; PC, phage cocktail; AVE, *Aloe vera* extract; FCAP, formulated cosmetic gel + *Aloe vera* extract + phage cocktail.

a–d: Differences between the study sites indicated by different letters are statistically significant (*P* < 0.05). Treatments with different letters are significantly different from each other, with “a” representing the Control group.

## Discussion

*Pseudomonas aeruginosa* and* Staphylococcus aureus* are pathogenic bacteria known for their ability to form strong biofilms, which can lead to chronic infections, impaired wound healing, and antibiotic resistance^34^. European guidelines advise against the use of antibiotics for treating co-infections of *P. aeruginosa* and* S. aureus*, and topical antibiotics are not recommended for this combination of pathogens [− 34]. This study aimed to isolate *P. aeruginosa* and* S. aureus* from wounds and explore the use of bacteriophages as an alternative treatment strategy. The bacterial isolates were identified using the VITEK2 system, a sophisticated platform for phenotypic identification that employs colorimetric reagent cards and advanced hardware and software^35^.

*Aloe vera*, a succulent plant widely recognized for its medicinal properties, has been utilized for centuries to treat a broad spectrum of ailments^36,37^. This study explores the antibacterial activity of *Aloe vera* gel extracts against common pathogens responsible for skin infections. In the present studies, the extract was tested against *Staphylococcus aureus* and *pseudomonas aeruginosa*, all of which showed positive results demonstrated that *Aloe vera* gel extracts exhibited significant inhibitory effects on all tested bacterial strains. These findings suggest that *Aloe vera* possesses potent antibacterial properties, making it a promising natural alternative for the treatment of skin infections.

The skin cosmetic with *Aloe* extract gel included ingredients such as stearic acid, cetyl alcohol, beeswax, ceteareth-20, paraffin oil, carbomer triethanolamine, methylparaben, propylparaben, perfume, and distilled water, as outlined by^27^. The addition of *Aloe vera* extract to the cosmetics enhances preservation and exhibits potent antibiofilm effects against *S. aureus* and* P. aeruginosa*, thereby ensuring consumer safety and extending shelf life^38^. The presence of chemical preservatives and antimicrobials in the cosmetic and *Aloe* extract, including ascorbic acid, p-coumaric acid, pyrocatechol, and cinnamic acid, each with distinct mechanisms of action against *S. aureus* and* P. aeruginosa*, contributes to its efficacy.

Previous results showed that *Aloe* gel inhibited the growth of *P. aeruginosa* and *Candida albicans*^39^. Other constituents of *Aloe vera* have demonstrated significant antimicrobial activity against bacteria, viruses, fungi, and yeasts. These findings align with^40^, who reported that Aloe vera exhibited broad-spectrum antibacterial properties against *E. coli, S. aureus, Bacillus subtilis, Streptococcus pyogenes, Bacillus cereus, P. aeruginosa, Salmonella typhi,* and *Klebsiella pneumonia*. This broad-spectrum activity is attributed to compounds like coumaric acid, pyrocatechol, ascorbic acid, and cinnamic acid, identified using thin-layer chromatography (TLC) and known to work synergistically. Additionally, the antibacterial effects of anthraquinones and saponins were noted.

Recent studies, such as those by^41^, have highlighted *Aloe vera* as a versatile natural cosmetic ingredient. *Aloe vera* is easily accessible and widely cultivated in Indonesia, offering numerous health benefits, including anti-inflammatory, antibiotic, antiseptic, antibacterial, antiviral, and anti-infective properties. It contains polysaccharides, essential amino acids, protein-degrading enzymes, folic acid, inositol, vitamins A, B12, and E, as well as minerals like calcium, magnesium, potassium, sodium, iron, zinc, and chromium. *Aloe vera* is used in various cosmetic products, including shampoo, cream bath, liquid soap, lotion, and lip balm.

According to^40^, *Aloe vera* extract exhibits significant antibacterial properties, effectively killing, inhibiting, or eradicating *S. aureus* growth. The antimicrobial effects of *Aloe vera* extract were particularly pronounced against Gram-positive bacteria like *S. aureus*.

Research suggests that phage-replacement therapy is a promising approach for managing pathogenic bacteria in dermatologic diseases due to the specificity of phages for their target bacteria. This need is especially urgent given the rise in antibiotic resistance, as noted by^42,43^.

The formulated cosmetic gel and bacteriophage populations (FCGAVE) protect the phage populations due to the inclusion of antioxidant substances in the *Aloe* extract gel^44,45^ preventing damage from atmospheric ultraviolet (UV) exposure. Antimicrobial and antioxidant preservatives further suppress oxidation processes and prevent free radical formation, extending the product’s shelf life and usage time beyond that of the cosmetic product without *Aloe* extract gel.

Bacteriophage therapy, although initially overshadowed by antibiotics, is regaining attention as antibiotic resistance continues to rise. Studies, including those by^46^, highlight the potential of bacteriophages as low-risk, high-efficiency alternatives for treating antibiotic-resistant infections.

Isolated bacteriophages specific to *S. aureus* and *P. aeruginosa* were detected in infected skin using the overlay technique and identified based on plaque morphology and morphotypes. These phages, characterized by their long, short, or curled non-contractile tails (200.3 to 245.5 nm in length and 15.4 to 18.5 nm in width) and head diameters (65.2 to 75.5 nm), were classified into the families *Myoviridae, Podoviridae, and Siphoviridae* based on the old ICTV classification, but with the new ICTV classification, they classified as *Straboviridae, Drexlerviridae,* and *Autographiviridae*.

The stability of bacteriophage populations at room temperature was assessed in a host-free environment. The combination of bacteriophages with the formulated cosmetic *Aloe vera* gel significantly increased the longevity of the phages against *S. aureus* and* P. aeruginosa* for over three months at room temperature. These phages also demonstrated stability across a pH range of 4–10, and under UV irradiation up to 45 cm for 30, 60, 90, and 120 min, and incubation at 37 °C for 30 min. Phage survival was determined using the plaque assay technique with *S. aureus* and* P. aeruginosa* as indicator hosts. Ensuring the stability of bacteriophages during storage is a crucial aspect of their application, which should be clearly defined for selected phages.

In previous studies, the amount of unabsorbed phage was estimated using the double-layer agar technique. The latent period of the phage, defined as the time until substantial amounts of phage particles first appeared, ranged from 36 to 45 min. This indicates that both the phage adsorption time and latent period were brief. These traits, along with the high production of lytic phages in host bacterial cells, are essential for effective phage therapy.

Isolates of *S. aureus* and* P. aeruginosa* demonstrated rapid, robust growth, colonization, and biofilm formation. *S. aureus* and* P. aeruginosa* cells attach to surfaces and become immobilized in a polysaccharide matrix to form biofilms. Due to the protective extracellular polymeric substance (EPS) layer, biofilm-associated *S. aureus* and* P. aeruginosa* are more resistant to sanitation treatments than their free-floating counterparts. The biofilm hinders the penetration of chemicals, rendering sanitizers less effective.

The cosmetic gel containing *Aloe vera* extract (CGAVE), the phage cocktail (PC), successfully inhibited biofilm formation of *S. aureus* and *P. aeruginosa*. The results demonstrated that the combination of phages with the cosmetic gel exhibited strong antibiofilm activity, outperforming the gel or *Aloe vera* extract alone. These findings suggest that a cosmetic formulation incorporating phages can provide additional protection, especially when applied at optimal times^23^.

Current research focuses on creating anti-adhesive surfaces to reduce bacterial biofilm formation or prevent bacterial adhesion. Alternative physical and chemical treatments, as well as antimicrobial photodynamic therapy, are being explored. Another promising approach involves using phage populations to eradicate bacterial biofilms. Phages are safe for humans, animals, and plants as they specifically target bacteria without infecting other organisms. Virulent (lytic) phages replicate within bacterial cells and release progeny phages. Antibiotic resistance is a growing problem globally, including in Eastern Europe, where phages have been used as an alternative to antibiotics to treat pathogenic bacteria. Phages encode lytic proteins such as endolysins and virion-associated peptidoglycan hydrolases (VAPGHs), as well as polysaccharide polymerase, making them effective antibiofilm agents^11^. Scientific studies have investigated the use of phages to inhibit biofilm formation by bacteria such as *Pseudomonas* spp., *S. epidermidis*, and *E. coli* O157.

This research highlights the potential of combining *Aloe vera* gel with phage populations to enhance the effectiveness of phage therapies, offering a novel approach to treating antibiotic-resistant bacterial infections and improving skin health.

## Conclusion

The efficacy of combining bacteriophages and *Aloe vera* extract as antibacterial agents demonstrates significant potential in combating pathogenic bacteria, particularly those that are multidrug-resistant. Bacteriophages, with their specificity and ability to lyse bacterial cells, offer a promising alternative to traditional antibiotics, especially in the face of increasing antibiotic resistance. *Aloe vera* extract, renowned for its antimicrobial properties, enhances this therapeutic approach through its bioactive compounds, which inhibit bacterial growth and biofilm formation. This synergistic application not only broadens the antimicrobial spectrum but also improves the overall effectiveness of treatment. The *Aloe vera* extract provides a conducive medium for bacteriophage stability and activity, ensuring prolonged and effective bacterial eradication. In summary, the integration of bacteriophages and *Aloe vera* extract into a single therapeutic regimen offers a novel and potent strategy to address the challenges posed by antibiotic-resistant bacteria. This approach not only provides an effective means of bacterial control but also promotes better skin health, highlighting its potential as a valuable tool in modern antimicrobial therapy.

## Data Availability

The authors declare that the article contains all the data established and analyzed during this investigation.
